# CHRONOLOGICAL AGE MODIFIES THE RELATIONSHIP BETWEEN HEALTH COMORBIDITIES AND PERCEIVED HEALTH STATUS

**DOI:** 10.1093/geroni/igae098.2247

**Published:** 2024-12-31

**Authors:** Brian Downer, Jose Cabrero Castro, Anita Brandt

**Affiliations:** The University of Texas Medical Branch, Galveston, Texas, United States; The University of Texas Medical Branch, Galveston, Texas, United States; The University of Texas Medical Branch, Galveston, Texas, United States

## Abstract

Societal norms perpetuate the notion that declining health is a normal part of aging. However, it is unclear whether chronological age modifies the relationship between health status and how middle-aged and older adults perceive their health relative to others their age. This analysis used data from 13,246 participants from the 2021 wave of the Mexican Health and Aging Study. Participants were asked if their health was better, the same, or worse than people their age. We grouped participants into four age groups (50-59, 60-69, 70-79, >80) and the number of health conditions (0, 1, 2, 3, >4). We used logistic regression models to estimate predicted probabilities of having better perceived health, adjusting for demographic characteristics, life satisfaction, depressive symptoms, and having been diagnosed with COVID-19. Nearly half (44.5%) of participants said their health was better than people their age, 46.1% said their health was the same, and 9.3% said their health was worse. The probability of better perceived health decreased with more health conditions and increased with age. The interaction between age and health conditions was statistically significant (p=0.009). The probability of having better perceived health decreased with more health conditions for participants aged 50-59, 60-69, and 70-79. Among participants aged >80, the probability for better perceived health was similar for participants with 0, 1, 2, and 3 health conditions but was lower for participants with >4 health conditions. Our findings suggest that older adults in Mexico have positive perceptions of their health despite a high prevalence of comorbid conditions.

